# The Transcription of Flagella of Enteropathogenic *Escherichia coli* O127:H6 Is Activated in Response to Environmental and Nutritional Signals

**DOI:** 10.3390/microorganisms10040792

**Published:** 2022-04-09

**Authors:** Fabiola Avelino-Flores, Jorge Soria-Bustos, Zeus Saldaña-Ahuactzi, Ygnacio Martínez-Laguna, Jorge A. Yañez-Santos, María L. Cedillo-Ramírez, Jorge A. Girón

**Affiliations:** 1 Centro de Investigación en Ciencias Microbiológicas, Benemérita Universidad Autónoma de Puebla, Puebla 72570, Mexico; avelinofloresf@yahoo.com (F.A.-F.); ignacio.martinez@correo.buap.mx (Y.M.-L.); 2 Instituto de Ciencias de la Salud, Universidad Autónoma del Estado de Hidalgo, Pachuca 42160, Mexico; jorge_s41@hotmail.com; 3 Paul G. Allen School for Global Health, College of Veterinary Medicine, Washington State University, Pullman, WA 99164, USA; zeus_drago@hotmail.com; 4 Facultad de Estomatología, Benemérita Universidad Autónoma de Puebla, Puebla 72410, Mexico; j_antonio_yanez@yahoo.com; 5 Centro de Detección Biomolecular, Benemérita Universidad Autónoma de Puebla, Puebla 72592, Mexico; lcedil9@gmail.com

**Keywords:** flagella, enteropathogenic, *Escherichia coli*, gut mucosa, virulence

## Abstract

The flagella of enteropathogenic *Escherichia coli* (EPEC) O127:H6 E2348/69 mediate adherence to host proteins and epithelial cells. What environmental and nutritional signals trigger or down-regulate flagella expression in EPEC are largely unknown. In this study, we analyzed the influence of pH, oxygen tension, cationic and anionic salts (including bile salt), carbon and nitrogen sources, and catecholamines on the expression of the flagellin gene (*fliC)* of E2348/69. We found that sodium bicarbonate, which has been shown to induce the expression of type III secretion effectors, down-regulated flagella expression, explaining why E2348/69 shows reduced motility and flagellation when growing in Dulbecco’s Minimal Essential Medium (DMEM). Further, growth under a 5% carbon dioxide atmosphere, in DMEM adjusted to pH 8.2, in M9 minimal medium supplemented with 80 mM glucose or sucrose, and in DMEM containing 150 mM sodium chloride, 0.1% sodium deoxycholate, or 30 µM epinephrine significantly enhanced *fliC* transcription to different levels in comparison to growth in DMEM alone. When EPEC was grown in the presence of HeLa cells or in supernatants of cultured HeLa cells, high levels (4-fold increase) of *fliC* transcription were detected in comparison to growth in DMEM alone. Our data suggest that nutritional and host signals that EPEC may encounter in the intestinal niche activate *fliC* expression in order to favor motility and host colonization.

## 1. Introduction

Enteropathogenic *Escherichia coli* (EPEC) is a world-wide cause of acute infantile diarrhea. EPEC attaches to host epithelial cells using a repertoire of adhesins including bundle-forming pilus (BFP), the outer membrane protein intimin, flagella, curli, and *E. coli* common pilus (ECP) [1,2,3,4]. In the small bowel, EPEC causes attaching and effacing (AE) lesions that lead to the destruction of the intestinal microvilli, opening of tight junctions, and release of water and electrolytes [1]. The gene products responsible for the production of the AE lesions are encoded in a chromosomal pathogenicity island called the locus of enterocyte effacement (LEE) [5]. 

The production of EPEC virulence factors is under the control of virulence-gene regulators (*perABC*, *ler*, *grlA*, and *grlR*) and an array of global regulators including IHF, BipA, H-NS, RpoS, Fis, QseBC, and others [6,7,8,9].

The bacterial flagellum is a surface organelle that promotes motility, and it is also involved in virulence-associated properties in a wide range of pathogenic bacteria. These properties include adherence, invasion, colonization, hemagglutination, biofilm formation, binding to host proteins, induction of proinflammatory responses via TLR5, and translocation of virulence molecules [2,10,11,12,13,14,15,16,17]. The synthesis, assembly, and function of the *E. coli* and *Salmonella enterica* flagella are under the control of a flagellar regulon that comprises more than 50 genes divided among at least 17 operons, among which *flhDC* is the master regulon that directs flagella expression [18,19,20,21,22]. Regulation of flagella is remarkably complex, involving many regulatory networks that respond to specific environmental conditions [23,24,25,26,27]. It is well known that the motility and production of flagella in *E. coli* K-12 and other bacteria are affected by growth conditions subjected to different pH, temperature, osmolarity, organic nutrients, bile, and salts [24,28,29,30,31,32,33]. 

The flagella of EPEC O127:H6 E2348/69 were previously shown to mediate adherence to epithelial cells and to bind to bovine mucus and host proteins such as mucins and extracellular matrix proteins [2,34]. While the production of most virulence factors of EPEC (BFP, intimin, type 3 secretion system [T3SS] and its effectors) is induced in the presence of epithelial cells or upon growth in Dulbecco’s Minimal Essential Medium (DMEM), the production of flagella and motility is abrogated when EPEC grows in this tissue culture medium. In contrast, bacterial growth in Luria-Bertani (LB) medium favors the manifestation of these phenotypes [2]. The environmental, nutritional, and host signals that trigger flagella production in EPEC are largely unknown. In this study, we aimed to quantitatively determine the influence of pH, oxygen tension, cationic and anionic salts, carbon and nitrogen sources, catecholamines, lysophosphatidic acid (LPA), and epithelial cells on the expression of the flagellin (*fliC)* gene of EPEC in vitro. The data from this investigation advance our knowledge on the signals that these bacteria may encounter in the niche of the intestinal mucosa to trigger flagella expression in order to favor motility and host colonization.

## 2. Materials and Methods

### 2.1. Bacterial Strains and Construction of the fliC::lacZ Transcriptional Fusion

Transcriptional fusions have been widely employed to analyze gene expression of numerous bacterial virulence factors [35,36,37,38,39]. Here, a transcriptional fusion consisting of the EPEC *fliC* promoter linked to a promoter-less *lacZ* reporter gene was constructed to monitor the expression of *fliC* in the EPEC O127:H6 strain E2348/69 under a variety of growth conditions. Primer FAV3 (5′-CGCGGATCCAAACGGTTAGCAATCGCCTG-3′), which was derived from the coding region of the EPEC *fliC* gene (www.sangerinstitute.org (accessed on 11 September 2021)), and FAV5 (5′-TCAAGCTTGGAACTTAAATCCAG-3′), derived from the coding region of the *fliD* gene, were used for amplification of the *fliC* promoter with *Pwo* polymerase using E2348/69 chromosomal DNA as the template. The amplicon was then ligated into plasmid pCR-Blunt II-TOPO yielding pFAV35. An *Eco*RI-digested fragment containing the *fliC* promoter from pFAV35 was ligated into the *Eco*RI site of the pRS551 vector [40], yielding pFAV36. The pRS551 vector contains the promoter-less *lacZ* reporter gene. The pFAV36 plasmid was transformed into E2348/69, and the transcriptional activity of *fliC* was monitored by measuring β-galactosidase activity, as previously described [40,41]. In all experiments, E2348/69 carrying the empty vector pRS551 was employed as a negative control.

### 2.2. Culture Conditions and β-Galactosidase Assays

EPEC E2348/69 containing the *fliC::lacZ* fusion or the empty vector was grown with shaking for 21 h at 37 °C, diluted 1:50 in fresh Luria-Bertani (LB containing 10 g/L NaCl) (Sigma Aldrich, St. Louis, MI, USA) or low-glucose DMEM (Gibco) and M9 minimal medium (25.6 g/L of Na_2_HPO_4_, 6 g/L of KH_2_PO_4_, 1 g/L of NaCl, and 2 g/L of NH_4_Cl) (Gibco). These media were reconstituted with different compounds at various concentrations and used to measure *fliC* expression after growth of EPEC to an OD_600_ of 0.65–0.70 at 37 °C. The pH of the supplemented media was adjusted to pH 7.2 before growth [42]. Compounds of study included divalent cationic salts, ammonium and sodium salts, different carbon and nitrogen sources, and hormones (epinephrine and norepinephrine) used at physiological and non-physiological concentrations. Lysophosphatidic acid (LPA) (Avanti Polar Lipids) was used at 200 mM in LB-grown bacteria after stimulation for 10 min, 1 h, 2 h, or 3 h of incubation at 37 °C, as previously described [43]. The cultures were then diluted 1:5 in Z buffer (0.06 M Na_2_HPO_4_, 0.04 M NaH_2_PO_4_, 0.01 M KCl, 0.001 M MgSO_4_, and 0.05 M β-mercaptoethanol), and the β-galactosidase activity was assayed using the O-nitrophenyl-β-D-galactopyranoside (ONPG) substrate, as previously described [40]. The β-galactosidase experiments were repeated at least three times in triplicate for each condition tested. Basal β-galactosidase activities in LB or DMEM media alone were used as a reference for the analysis. 

### 2.3. Adherence to Epithelial Cells and Detection of Flagella by Immunofluorescence

HeLa cells were cultivated at 37 °C under a 5% CO_2_ atmosphere in polystyrene 24-well plates (Cellstar) containing glass coverslips, as previously described [2]. Briefly, cell monolayers at 80% confluency, were infected from 1 h to 6 h with 10 µL (10^7^ bacteria) of a bacterial suspension with an OD_600_ of 1.1 (10^9^ bacteria/mL) previously grown overnight in LB. The cells were washed with PBS to remove unbound bacteria and then fixed with 2% formalin/PBS for immunofluorescence. Primary rabbit anti-H6 antibodies were added for 1 h at a 1:3000 dilution in 10% normal horse serum. After washing, the cells were incubated for 1 h with secondary anti-rabbit IgG Alexa 488 fluor-conjugated antibodies diluted 1:3000. The cells were washed extensively and mounted in glycerol-PBS and visualized under UV light using a Zeiss Axiolab microscope.

### 2.4. Pre-Conditioned Medium

Monolayers of HeLa cells that had been extensively washed with PBS were incubated in DMEM without antibiotics or fetal bovine serum for 24–48 h. The supernatant from these cell cultures referred as to “pre-conditioned medium” (P-DMEM) was collected and the pH adjusted to 7.4 and filtered through a 0.2 µm membrane. The P-DMEM was supplemented with ampicillin and used to grow E2348/69 containing pRS551 or pFAV36 to quantitate the expression of flagellin. 

### 2.5. Motility Assays

Motility assays were performed in 0.3% agar plates containing tryptone media (1% tryptone) and reconstituted with different compounds. Briefly, the agar was spiked with an overnight culture using a needle and incubated at 37 °C. The level of motility was assessed by examining the radius of opacity as a result of bacterial swimming away from the inoculation point after 16 h of incubation as follows: +++: highly motile; ++: moderate motile; +: weak motile; -: non motile.

### 2.6. Statistical Analysis

All data were the averages of at least 3 independent experiments performed in triplicate. GraphPad Prism 9 software (GraphPad, San Diego, CA, USA) was used for statistical differences. One-way ANOVA followed by Tukey’s multiple comparison test and an unpaired Student’s *t* test was performed. A *p*-value ≤ 0.05 was considered statistically significant.

## 3. Results

### 3.1. Glucose Activates fliC Expression in EPEC

**Figure 1:**
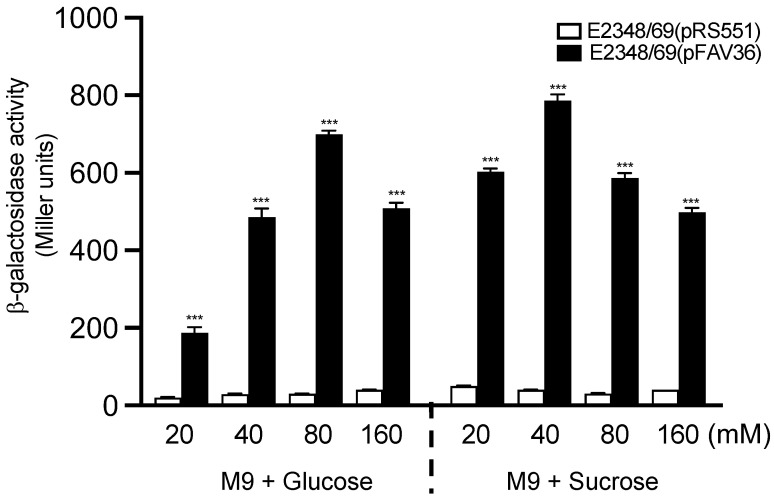
Effect of glucose and sucrose on *fliC* expression in EPEC. Activation of *fliC* transcription was measured in E2348/69 carrying pFAV36 (*fliC::lacZ* fusion) (black bars) and E2348/69(pRS551) (white bars) during growth in M9 minimal medium containing 20 mM to 160 mM of glucose (M9 + Glu) or sucrose (M9 + Suc). These data are the mean of at least three experiments performed in triplicate on different days. *** *p* < 0.001.

In the niche of the intestinal tract, there is a gradient of glucose concentration that diminishes from the small to the large intestine. In contrast, the concentration of ammonium, which is generated from protein degradation, increases from the small bowel towards the large bowel [44,45,46]. Thus, we were interested in determining if glucose had any effect on the expression of *fliC* in EPEC. We began this study by determining the effect of glucose on EPEC *fliC* expression in M9 minimal media supplemented with 20 mM to 160 mM glucose (M9 + Glu). In parallel assays, we also employed a second hexose, sucrose, as an alternate source of carbon during growth (M9 + Suc). A dose-dependent effect on *fliC* expression was noted for both sugars with maximum expression reached at 80 mM glucose (*p* < 0.001) and 40 mM sucrose (*p* < 0.001) (Figure 1). Sucrose at 20 mM induced a 3-fold increase in *fliC* expression as compared to 20 mM glucose. At a 40 mM concentration, sucrose induced *fliC* expression ~1.55-fold higher than glucose at 40 mM. *fliC* expression began to decline when glucose and sucrose were present over 80 mM and 40 mM, respectively. Thus, flagella activation was not glucose-specific since sucrose also induced *fliC* transcription.

### 3.2. Role of Sodium Bicarbonate in Flagella Expression

**Figure 2:**
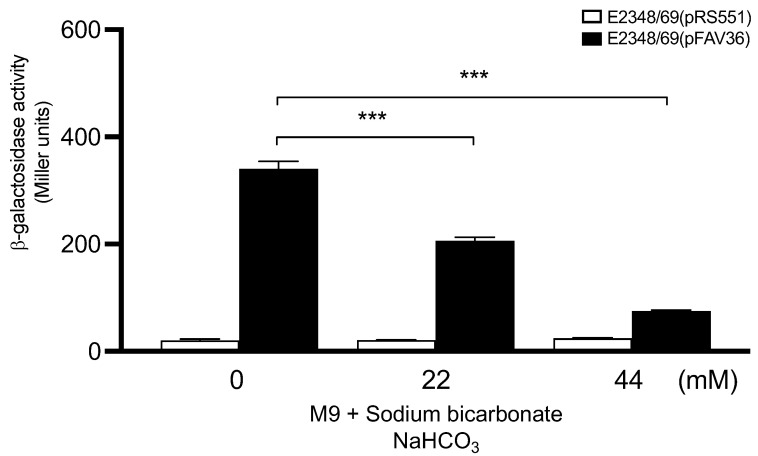
Effect of sodium bicarbonate on *fliC* expression in EPEC. Sodium bicarbonate (22–44 mM) was added to M9 minimal medium to determine its effect on *fliC* transcription. These data are the mean of at least three experiments performed in triplicate on different days. The negative control for background *lacZ* activity was E2348/69(pRS551) (white bars). *** *p* < 0.001.

Sodium bicarbonate is a component of DMEM and was reported to stimulate the expression of genes contained in the LEE region of EHEC O157:H7 [47]. EPEC also contains a homolog of the EHEC LEE region [48]. That report led us to determine whether the addition of sodium bicarbonate to M9 minimal medium would affect *fliC* expression in EPEC. In contrast to the bicarbonate-mediated activation of EHEC LEE-encoded genes previously reported, we found that the presence of this compound reduced, in a dose–response manner, the expression of *fliC* transcription in EPEC (Figure 2). Analysis of the composition of the DMEM employed revealed that bicarbonate was present at 44 mM, a concentration at which we saw significant reduction of *fliC* transcriptional activation. This result may explain, at least in part, the negative regulation exerted on flagellation and motility previously reported in EPEC E2348/69 growing in DMEM [2].

### 3.3. Modulation of fliC Expression in EPEC by Ammonium

**Figure 3:**
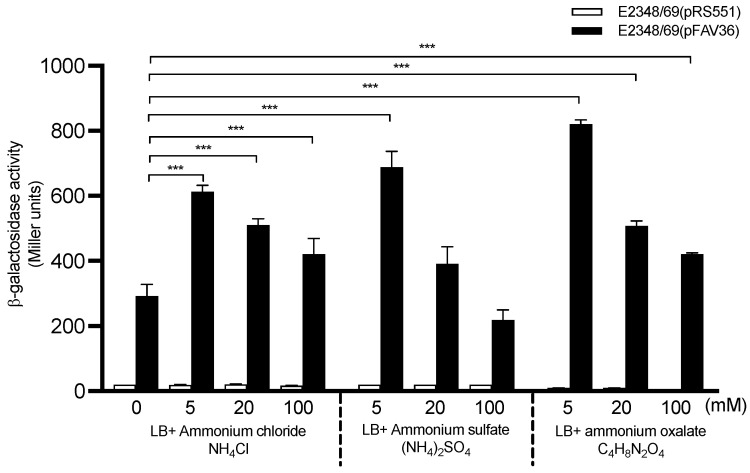
Effect of ammonium salts on *fliC* expression in EPEC. Different sources of ammonium from 5–100 mM were added to LB to determine its effect on *fliC* transcription. These data are the mean of at least three experiments performed in triplicate on different days. The negative control for background *lacZ* activity was E2348/69(pRS551) (white bars). *** *p* < 0.001.

Ammonium was previously shown to influence the production of BFP in the EPEC O111:NM strain B171 [49]. Here, we sought to study the effect of ammonium in *fliC* expression in EPEC E2348/69 by using three ammonium salts (sulfate, chloride, and oxalate) added at 5 mM, 20 mM, and 100 mM to M9 minimal medium. We did not see any effect of these salts on *fliC* expression when added to M9 medium (data not shown); thus, we studied their effect in LB-grown bacteria. Among the three salts tested, ammonium oxalate showed the highest levels of *fliC* transcription (Figure 3). At 5 mM of ammonium salts, a 2.4-fold increase in *fliC* transcription was recorded compared to growth in LB without ammonium (*p* < 0.001). We also studied the effect of ammonium in DMEM-grown bacteria; however, the β-galactosidase activity found in DMEM with ammonium sulfate at any given concentration was even less than in DMEM alone (data not shown), suggesting that growth in DMEM with ammonium sulfate had a negative effect on *fliC* expression. 

### 3.4. Effect of Sodium Chloride and Sodium Bisulfite on Flagella Expression

**Figure 4:**
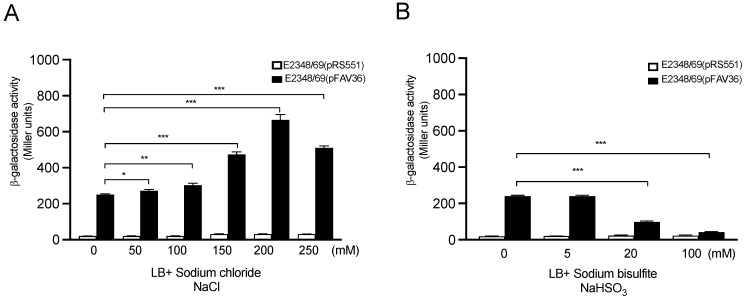
Effect of sodium chloride (**A**) and sodium bisulfite (**B**) on *fliC* expression in EPEC. Different sources of sodium from 50–250 mM were added to LB to determine its effect on *fliC* transcription. These data are the mean of at least three experiments performed in triplicate on different days. The negative control for background *lacZ* activity was E2348/69(pRS551) (white bars). * *p* < 0.05; ** *p* < 0.01; *** *p* < 0.001.

Sodium (chloride and bisulfite) salts were used for the assessment of *fliC* transcription. NaCl triggered dose-dependent *fliC* expression when used at 50 mM to 250 mM in LB with a peak at 200 mM, which represents a 3-fold increase compared to LB without NaCl (*p* < 0.001) (Figure 4A). Sodium bisulfite was previously shown to induce the synthesis of BFP in EPEC strains [50]. However, here, we found that sodium bisulfite showed an inhibitory effect on flagellin expression (Figure 4B and Table 1). 

### 3.5. Influence of Divalent Cationic Salts on Flagella Expression

**Figure 5:**
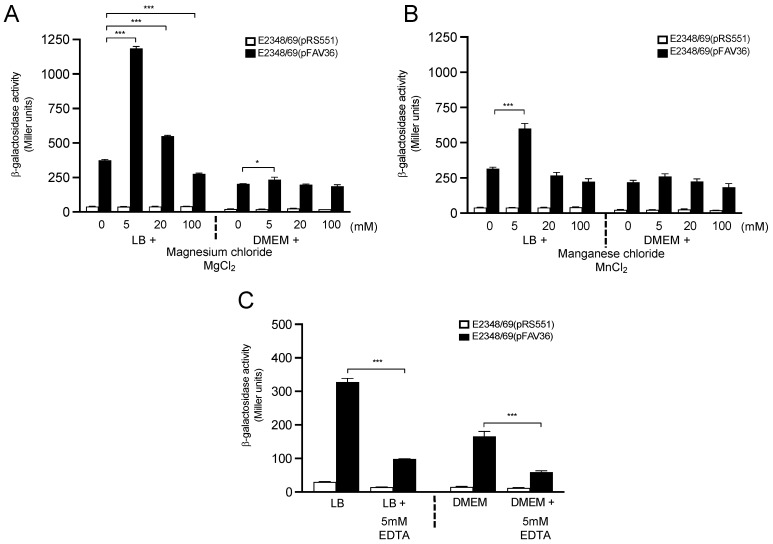
Effect of magnesium and manganese divalent cations on *fliC* expression in EPEC. (**A**) Magnesium and (**B**) manganese chloride were added at 5–100 mM to LB or DMEM to measure *fliC* expression in E2348/69(pFAV36) (black bars) and E2348/69(pRS551) (white bars). (**C**) Effect of the addition of 5 mM EDTA to LB or DMEM on *fliC* expression. These data are the mean of at least three experiments performed in triplicate. * *p* < 0.05; *** *p* < 0.001.

Divalent cations are central elements in the integrity of the cell membrane, as well as in many cellular functions. In EPEC, the expression of BFP and T3SS-dependent effector proteins is influenced by the presence of divalent cations (Ca^2+^ and Mg^2+^) in the growth media [49,51]. Growth in DMEM triggers the production of most virulence factors in EPEC [49,51,52]. Therefore, we employed divalent cationic salts MgCl_2_ and MnCl_2_ at various concentrations (5 mM, 20 mM, and 100 mM) to define their role in *fliC* transcription when added in parallel to LB and DMEM growth media. When 5 mM MgCl_2_ was added to LB, *fliC* expression was increased 4-fold compared to growth in LB alone (*p* < 0.001). However, a significant effect on *fliC* expression was noted only when bacteria were grown in DMEM supplemented with 5 mM of MgCl_2_ (*p* < 0.05) (Figure 5A,B). Similar results were obtained when magnesium sulfate was employed, suggesting that it is the magnesium cation, and not the sulfate or chloride anions, that triggers *fliC* expression (data not shown). When 5 mM MnCl_2_ was used in LB, increased *fliC* transcription (1.9-fold, *p* < 0.05) was observed (Figure 5B). The presence of calcium chloride did not significantly activate β-galactosidase activity; however, bacterial motility was increased when the salt was added to DMEM (Table 1). Thus, Mg^2+^ seems to be the most important of these divalent cations in activating flagella expression. To further support a role for divalent cations in *fliC* transcription, 5 mM EDTA (chelator of divalent cations) was added to the LB and DMEM growth media. As predicted, a significant reduction in *fliC* expression was seen with the addition of EDTA to LB (~70%) (*p* < 0.001) and DMEM (64%) (*p* < 0.001) (Figure 5C), stressing the need for divalent cations in flagellin expression.

### 3.6. Host Gut Signals Influence Expression of EPEC fliC

**Figure 6:**
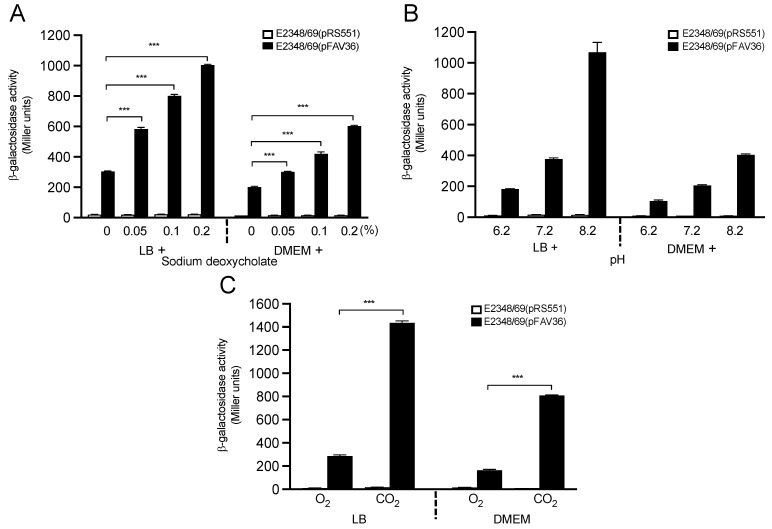
Putative host signals that activate *fliC* expression in EPEC. The effect of sodium deoxycholate (0.05–0.2%) on *fliC* expression was studied in LB and DMEM (**A**). LB and DMEM were adjusted to the indicated pH and tested for β-galactosidase activation (**B**). Note the significant increase of *fliC* transcription in both media. Bacteria were incubated in LB or DMEM with aeration or under a 5% carbon dioxide atmosphere. Note the increased expression of *fliC* (**C**). These data are the mean of at least three experiments performed in triplicate. The negative control for background *lacZ* activity was E2348/69(pRS551) (white bars). *** *p* < 0.001.

Host signals that prevail at the site of bacterial colonization in the intestine, such as bile acids, hormones, oxygen, carbon dioxide, and pH, determine in part which virulence-associated products are expressed in vivo. Thus, we sought to evaluate the role of these intestinal cues in *fliC* expression in vitro after growth in LB and DMEM media. We found that the presence of sodium deoxycholate (bile) in both LB and DMEM was associated with a dose-dependent β-galactosidase activity. At 0.2% sodium deoxycholate, a 3.6-fold and 3-fold increase in transcription were detected in LB and DMEM (*p* < 0.001), respectively, in comparison to the medium without deoxycholate (Figure 6A). At this physiological concentration, bile salt may presumptively act as an activator of flagellin synthesis in the small intestine. 

In their passage from the stomach to and through the small and large intestines, bacteria experience a pH gradient from low-to-high. Thus, β-galactosidase activity was also measured in EPEC bacteria growing in LB and DMEM media adjusted to a pH range of 6.2 to 8.2 [43]. The pH of the media was measured before and after growth to ensure that the pH did not change by more than 0.05 pH units (data not shown). The optimal pH for activation of EPEC *fliC* transcription was 8.2 in both LB and DMEM, whereas the β-galactosidase activity was significantly lower at pH 6.2 and pH 7.2 (Figure 6B). 

In the lumen of the large intestine, facultative anaerobic bacteria such as *E. coli* live under an atmosphere of reduced oxygen tension, which could in turn act as a signal to activate genes required for intestinal colonization. Thus, we studied *fliC* transcription in bacteria grown with aeration or in a 5% carbon dioxide atmosphere. We found that the β-galactosidase activity was 5-fold increased after growth in both LB (*p* < 0.001) and in DMEM (*p* < 0.001) under a 5% carbon dioxide atmosphere compared to growth under aeration. Up to this point, carbon dioxide was the only element, amongst all compounds and conditions tested, that triggered the highest level of *fliC* expression in bacteria grown in DMEM (Figure 6C). This result is conceivably significant considering that the oxygen tension in the small intestine, the site of EPEC infection and colonization, is reduced relative to carbon dioxide [3].

### 3.7. The Presence of Epithelial Cells Triggers Flagella Expression in EPEC

**Figure 7:**
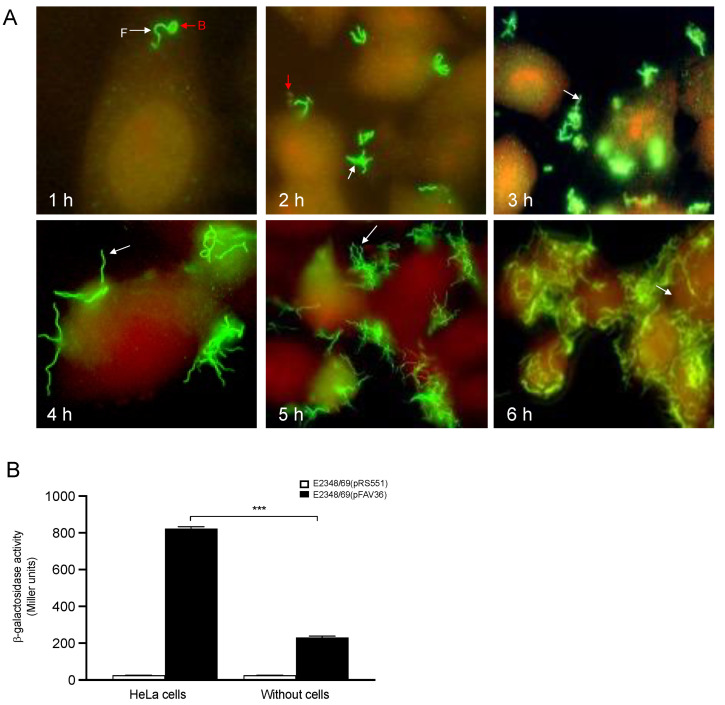
Host epithelial cells activate flagella expression and the kinetics of production of flagella by EPEC adhering to HeLa cells. HeLa cells were infected with E2348/69 from 1 h to 6 h and then reacted with anti-H6 antibodies (**A**). The production of H6 flagella was monitored by immunofluorescence. Note the time- and bacterial-concentration-dependent increasing production of flagella. *fliC* transcription was measured in the presence or absence of epithelial cells (**B**). These data are the mean of at least three experiments performed in triplicate. The negative control for background *lacZ* activity was E2348/69(pRS551) (white bars). F: flagella (white arrows); B: bacteria (red arrows). *** *p* < 0.001.

We monitored time-dependent (1–6 h) production of flagella by bacteria adhering to cultured HeLa cell monolayers by immunofluorescence using anti-flagella H6 antibodies [2]. At 1 h of infection, only a few bacteria adhered to the epithelial cells. As the time of infection increased, so did the number of bacteria attached to the cell monolayer. Interestingly, within 6 h of infection, the bacteria did not shut off flagella production. Instead, they continued to produce flagella as the adhering bacteria multiplied on the surface of the eukaryotic cells (Figure 7A). In contrast to these observations, in *S. enterica* and *B. bronchiseptica*, the synthesis of flagella was turned off once the bacteria were programmed to penetrate host epithelial cells [53,54]. In addition, transcription of *fliC* was measured in the presence and absence of epithelial cells. HeLa cells were infected with EPEC for 3 h, after which the transcription of flagella was determined using the β-galactosidase assay. *fliC* transcriptional activation in E2348/69 was increased 3.5-fold in the presence of HeLa cells compared to growth in DMEM without HeLa cells (Figure 7B), supporting our hypothesis that eukaryotic cells or a cellular-secreted product trigger flagella expression in EPEC [2].

### 3.8. Role of Endocrine Adrenergic Molecules in EPEC fliC Expression

**Figure 8:**
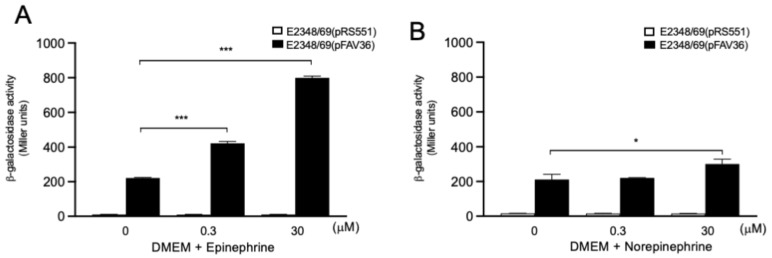
Role of catecholamines in *fliC* expression in EPEC. β-galactosidase activity was measured in DMEM containing 0–30 µM of epinephrine or norepinephrine (**A**,**B**). These data are the mean of at least three experiments performed in triplicate. The negative control for background *lacZ* activity was E2348/69(pRS551) (white bars). * *p* < 0.05; *** *p* < 0.001.

Catecholamines such as epinephrine and norepinephrine were previously shown to enhance the growth of commensal and pathogenic *E. coli* and to enhance virulence-associated properties of some *E. coli* pathogroups [55]. Regarding expression flagella and motility, it was reported that epinephrine and norepinephrine activate the expression of flagellar genes in *E. coli* O157:H7 [56,57]. In the present study, we tested epinephrine and norepinephrine as potential inducers of *fliC* transcription in EPEC. Bacteria were grown aerobically in DMEM supplemented with two concentrations (0.3 µM and 30 µM) of these molecules. We chose DMEM for these experiments because *fliC* transcription is considerably reduced in DMEM compared to LB, allowing us to better determine any positive effect on expression. The presence of epinephrine significantly enhanced (*p* < 0.001) the expression of EPEC flagella at the two different concentrations employed with a 3.6- and 1.9-fold increase, respectively (Figure 8A). Norepinephrine showed a more discrete increase in *fliC* transcription at 30 µM (1.4-fold, *p* < 0.05), but did not increase the expression of *fliC* at 0.3 µM (Figure 8B).

### 3.9. Host-Cell-Produced Lysophospholipids Do Not Activate fliC Transcription in EPEC

A previous report showed that host-cell-produced lysophospholipids (lysophosphatidic acid (LPA) and lysophosphatidylcholine (LPC)) trigger the synthesis and secretion of flagellin by *S. enterica* serovar Typhi during infection of intestinal epithelial cells [42]. We were interested in determining if cellular lysophospholipids activate transcription of *fliC* in EPEC. Commercially available LPA used at 200 mM in LB-grown bacteria showed no apparent effect on the transcription of *fliC* after stimulation for 10 min, 1 h, 2 h, or 3 h of incubation at 37 °C (data not shown). These observations suggest that different host signals activate *fliC* in EPEC and pathogenic *Salmonella*.

### 3.10. Reconstitution of a Bacteriological Medium Optimal for Flagella Expression

**Figure 9:**
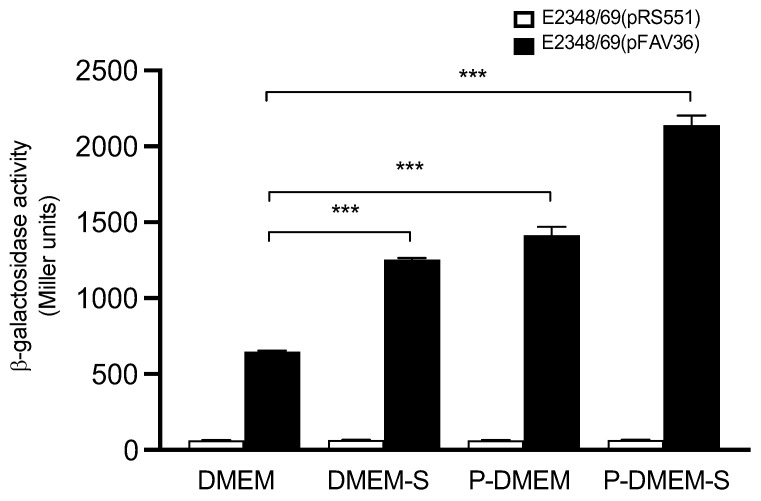
Reconstitution of media with *fliC* activating signals. Sodium chloride at 150 mM and 0.1% sodium deoxycholate were used in DMEM and P-DMEM (see the text for details) adjusted to pH 8.2 and then used to grow bacteria under a 5% carbon dioxide atmosphere. β-galactosidase activity was monitored as before. These data are the mean of at least three experiments performed in triplicate. The negative control for background *lacZ* activity was E2348/69(pRS551) (white bars). *** *p* < 0.001.

Based on the information obtained above, we attempted to formulate a growth medium containing the signals that induced the highest levels of *fliC* transcription. Since growth in DMEM showed poor induction of *fliC* expression, we adjusted the pH of this medium to 8.2 and added the key components that triggered flagellin expression: 150 mM sodium chloride and 0.1% sodium deoxycholate sodium. This supplemented medium is referred to as DMEM-S. We also obtained supernatants from cultured HeLa cells grown without antibiotics and filtered them to remove any cellular debris. This medium was labeled as pre-conditioned medium (P-DMEM). For comparison, we also supplemented P-DMEM with NaCl and bile salt to yield P-DMEM-S. Bacteria were incubated in DMEM, DMEM-S, P-DMEM, and P-DMEM-S media under a 5% carbon dioxide atmosphere at 37 °C. Maximal activation of *fliC* transcription was found in P-DMEM-S with an ~3-fold increase in comparison to DMEM alone (Figure 9), indicating that host cell signals play a significant role in *fliC* expression. 

### 3.11. Motility Assay

The motility of EPEC E2348/69 was assayed in DMEM containing 0.3% agar and supplemented with the compounds listed in Table 1. The results were in strong correlation with the *fliC* transcription data obtained with the β-galactosidase assay.

## 4. Discussion

Production and secretion of virulence factors in many bacterial pathogens are influenced by factors intrinsic to the host, the microenvironment, and the bacteria themselves. For example, the effect of the bacterial growth phase, the presence of carbon and nitrogen sources, salts, bile, hormones, pH, temperature, oxygen, and carbon dioxide may be important to trigger the expression of virulence genes in the host during infection or in extra-host environmental niches [23,58,59,60,61,62,63]. In EPEC, the environmental and nutritional signals that modulate the expression of BFP and other virulence factors have been previously reported [49,51,64,65,66]. Generally, the growth of EPEC in DMEM at 37 °C, with or without epithelial cells, allows the production of BFP and LEE-encoded products and the secretion of T3SS-associated effector molecules. The majority of studies regarding the signals that regulate flagella expression and motility have been conducted in *Salmonella* and laboratory *E. coli* strains [27,28,67,68,69]. Our previously published data suggested that environmental, nutritional, and host signals modulate *fliC* expression in EPEC, at least in vitro [2]. What cues have an effect on flagella production during colonization of the human gut by EPEC remains largely unknown. Thus, to learn about the chemical and biological nature of the signals that might be present in the intestinal tract and that trigger flagella production, we measured the transcriptional activation of *fliC* in E2348/69 propagated in a wide range of laboratory growth conditions.

Previously, we showed that the EPEC strain E2348/69 produces flagella upon growth in LB, whereas flagella production and motility are greatly diminished in DMEM. Interestingly, however, the bacteria were able to regain these phenotypes upon contact with cultured epithelial cells replicating in DMEM or when growing in HeLa cell supernatants, suggesting that host cell factors overcome the negative regulation exerted by DMEM components [2]. Some reports have shown that bicarbonate ion stimulates the expression of virulence genes [47,70,71]. We studied *fliC* transcription in EPEC growing in LB without and with sodium bicarbonate at 22 mM and 44 mM. Converse to the effect that bicarbonate has on the expression of LEE-encoded genes in EHEC, we saw a dose–response negative effect on flagella expression in EPEC. This negative effect may explain why the production of flagella and motility in EPEC are down-regulated when growing in DMEM, which contains 44 mM bicarbonate. In support of this idea, we found that bicarbonate might be such a component, since its addition to LB also inhibited *fliC* expression.

The influence of different carbohydrates on flagella and motility in *E. coli* K-12 has been investigated previously. Glucose was shown to inhibit motility at 0.01 M, and it was suggested that this negative effect on flagellation was through catabolic repression [28,68]. More recently, increased expression of *fliC* in adherent-invasive *E. coli* growing in glucose-supplemented media was shown [72]. In the present study, we found that the presence of glucose and sucrose in LB at the concentration range of 20 mM to 160 mM increased EPEC flagellin gene expression. These data suggest that the flagella in *E. coli* K-12 and EPEC are regulated differently by glucose. Positive regulation by glucose may be related to enhanced availability of energy sources. It is well established that there is a high-to-low glucose gradient from the small to the large intestine [45]. Thus, the presence of glucose in the lumen of the small intestine would presumptively be important to initiate the synthesis of flagella, as these appendages are required for the bacteria to swim across the mucus layer that bathes target epithelial sites.

Divalent cations such as Ca^2+^, Mg^2+^, and Mn^2+^ are central to the stability and integrity of the cell membrane and are required in many cellular functions. Calcium and magnesium ions regulate virulence gene expression in *E. coli*, *Klebsiella*, *Yersinia*, and *Salmonella* species, respectively [60,62,73,74,75]. In EPEC, the expression of BFP and translocated T3SS-effector proteins is also influenced by the presence of Ca^2+^ and Mg^2+^ salts in the growth media [49,51,76,77,78]. We demonstrated that the presence of cations such as Mg^2+^ and Mn^2+^ increased flagellin expression, but did not trigger motility. In favor of this notion, we showed that this effect was reversed by the presence of the chelating agent EDTA. In contrast, Ca^2+^ did not activate flagella expression, but favored motility. 

The concentration and availability of oxygen and carbon dioxide are relative to the site of the small and large intestines. *E. coli* is a facultative anaerobic organism and, as such, may or may not need oxygen to grow. It is clear from different studies in the literature that oxygen and carbon dioxide directly affect the production of virulence factors by regulating gene expression or indirectly by altering the environmental pH [51,61,79,80]. In this study, carbon dioxide was one of the most important inducers of *fliC* expression; this result correlates well with our observations that *fliC* expression was increased at pH values higher than 7.4.

Bile salts are steroids with detergent properties, which help in fat digestion and absorption through the intestinal wall [81,82]. The most abundant of the bile salts in humans are cholate and deoxycholate, and they are normally conjugated with either glycine or taurine to give glycocholate or taurocholate, respectively. Bile was also shown to be important for the expression of virulence factors in *V. cholerae* [83,84,85]. However, in *Proteus mirabilis* and *E. coli* K-12, 0.1% sodium deoxycholate inhibited flagellation and motility [86]. We found that sodium deoxycholate at a physiological concentration of 0.1% increased *fliC* expression in EPEC. We speculate that this bile component is important during infection of the small intestine by EPEC. 

Earlier studies showed that during infection of intestinal epithelial cells, *S. typhi* secretes abundant pro-inflammatory flagellins upon activation by the host-cell-produced lysophospholipids (LPA) and LPC) [42]. In our study, we did not see an increase in the transcription of EPEC *fliC* or in the secretion of flagellin when LPA was used as the activator, suggesting that different host signals activate *fliC* expression in EPEC and pathogenic *Salmonella*.

The relationship between stress-related neuroendocrine hormones known as catecholamines, which include adrenaline (epinephrine) and noradrenaline (norepinephrine), and bacterial pathogenesis has been recognized for over half a century [87,88]. Increased levels of norepinephrine in the intestinal lumen play an important role in bacterial pathogenesis [89,90]. At high concentrations, norepinephrine has been shown to enhance the growth of commensal and pathogenic *E. coli* and to enhance the virulence properties of enterotoxigenic (ETEC) and *E. coli* O157:H7 [55,56,87,91]. Increased EHEC and adherent-invasive *E. coli* (AIEC) adherence to porcine colonic mucosa and Caco-2 cells, respectively, were noted after treatment with norepinephrine [20,92]. We found that catecholamines activated *fliC* expression in EPEC.

It is apparent that the relationship between flagellar and virulence gene regulation is tightly linked and that a complex interplay is occurring at the molecular level to coordinate multiple phenotypes as they are needed in the host niche. The importance of this interplay with regard to flagella regulation is underscored by the attenuation in the virulence of mutants with altered flagella expression. Understanding the environmental and molecular regulators that ultimately determine flagella gene expression and its coordinated regulation with virulence factors can provide insights into the significance of the production of flagella in the niche of the intestinal tract and their role in host colonization.

## Figures and Tables

**Table 1:** Correlation of *fliC* transcription and motility in EPEC.

Condition or Substance Added to DMEM	*fliC* Transcription	Motility
Unmodified DMEM	-	-
Ammonium sulfate 5 mM	-	-
Sodium chloride 200 mM	++	+
Sodium bisulfite 5 mM	-	-
Sodium bicarbonate 44 mM *	-	-
Magnesium chloride 5 mM	+	+
Magnesium sulfate 5 mM	+	+
Manganese chloride 5 mM	+	+
Calcium chloride 5 mM	+	+++
EDTA 5 mM	-	-
Sodium deoxycholate 0.2%	++	++
pH 6.2	-	-
pH 8.2	+	+
5% CO_2_	+++	+++
HeLa cells	+++	+++
Epinephrine 30 µM	++	+++
Norepinephrine 30 µM	+	+++
Lysophosphatidic acid 200 mM	-	-

(-) = 0–199 Miller units; (+) = 200–400 Miller units; (++) = 401–800 Miller units; (+++) = 801–1200 Miller units; (-) = non-motile; (+) = weakly motile; (++) = moderately motile; (+++) = highly motile. * M9 minimal medium.

## Data Availability

Not applicable.

## References

[1] Nataro J.P., Kaper J.B. (1998). Diarrheagenic *Escherichia coli*. Clin. Microbiol. Rev..

[2] Girón J.A., Torres A.G., Freer E., Kaper J.B. (2002). The flagella of enteropathogenic *Escherichia coli* mediate adherence to epithelial cells. Mol. Microbiol..

[3] Kaper J.B., Nataro J.P., Mobley H.L. (2004). Pathogenic *Escherichia coli*. Nat. Rev. Microbiol..

[4] Rendón M.A., Saldaña Z., Monteiro-Neto V., Erdem A.L., Vázquez A., Kaper J.B., Puente J.L., Girón J.A. (2007). Commensal and pathogenic *Escherichia coli* use a common adherence factor for epithelial cell colonization. Proc. Natl. Acad. Sci. USA.

[5] McDaniel T.K., Kaper J.B. (1997). A cloned pathogenicity island from enteropathogenic *Escherichia coli* confers the attaching and effacing phenotype on *E. coli* K-12. Mol. Microbiol..

[6] Gómez-Duarte O.G., Kaper J.B. (1995). A plasmid-encoded regulatory region activates chromosomal *eaeA* expression in enteropathogenic *Escherichia coli*. Infect. Immun..

[7] Ibarra J.A., Villalba M.I., Puente J.L. (2003). Identification of the DNA binding sites of PerA, the transcriptional activator of the *bfp* and *per* operons in enteropathogenic *Escherichia coli*. J. Bacteriol..

[8] Lara-Ochoa C., González-Lara F., Romero-González L.E., Jaramillo-Rodríguez J.B., Vázquez-Arellano S.I., Medrano-López A., Cedillo-Ramírez L., Martínez-Laguna Y., Girón J.A., Pérez-Rueda E. (2021). The transcriptional activator of the bfp operon in EPEC (PerA) interacts with the RNA polymerase alpha subunit. Sci. Rep..

[9] Bustamante V.H., Villalba M.I., García-Angulo V.A., Vázquez A., Martínez L.C., Jiménez R., Puente J.L. (2011). PerC and GrlA independently regulate Ler expression in enteropathogenic *Escherichia coli*. Mol. Microbiol..

[10] Allen-Vercoe E., Woodward M.J. (1999). The role of flagella, but not fimbriae, in the adherence of *Salmonella enterica* serotype Enteritidis to chick gut explant. J. Med. Microbiol..

[11] Chua K.L., Chan Y.Y., Gan Y.H. (2003). Flagella are virulence determinants of *Burkholderia pseudomallei*. Infect. Immun..

[12] Gavin R., Merino S., Altarriba M., Canals R., Shaw J.G., Tomas J.M. (2003). Lateral flagella are required for increased cell adherence, invasion and biofilm formation by *Aeromonas* spp.. FEMS Microbiol. Lett..

[13] Zhou X., Girón J.A., Torres A.G., Crawford J.A., Negrete E., Vogel S.N., Kaper J.B. (2003). Flagellin of enteropathogenic *Escherichia coli* stimulates interleukin-8 production in T84 cells. Infect. Immun..

[14] Xicohtencatl-Cortes J., Lyons S., Chaparro A.P., Hernandez D.R., Saldaña Z., Ledesma M.A., Rendón M.A., Gerwitz A.T., Klose K.E., Girón J.A. (2006). Identification of proinflammatory flagellin proteins in supernatants of *Vibrio cholerae* O1 by proteomics analysis. Mol. Cell. Proteom..

[15] Friedlander R.S., Vogel N., Aizenberg J. (2015). Role of flagella in adhesion of *Escherichia coli* to abiotic surfaces. Langmuir.

[16] Bente K., Mohammadinejad S., Charsooghi M.A., Bachmann F., Codutti A., Lefèvre C.T., Klumpp S., Faivre D. (2020). High-speed motility originates from cooperatively pushing and pulling flagella bundles in bilophotrichous bacteria. eLife.

[17] Horstmann J.A., Lunelli M., Cazzola H., Heidemann J., Kühne C., Steffen P., Szefs S., Rossi C., Lokareddy R.K., Wang C. (2020). Methylation of *Salmonella* Typhimurium flagella promotes bacterial adhesion and host cell invasion. Nat. Commun..

[18] Chilcott G.S., Hughes K.T. (2000). Coupling of flagellar gene expression to flagellar assembly in *Salmonella enterica* serovar typhimurium and *Escherichia coli*. Microbiol. Mol. Biol. Rev..

[19] Macnab R.M. (2003). How bacteria assemble flagella. Annu. Rev. Microbiol..

[20] Green B.T., Lyte M., Chen C., Xie Y., Casey M.A., Kulkarni-Narla A., Vulchanova L., Brown D.R. (2004). Adrenergic modulation of *Escherichia coli* O157:H7 adherence to the colonic mucosa. Am. J. Physiol. Gastrointest. Liver. Physiol..

[21] Mouslim C., Hughes K.T. (2014). The effect of cell growth phase on the regulatory cross-talk between flagellar and Spi1 virulence gene expression. PLoS Pathog..

[22] Albanna A., Sim M., Hoskisson P.A., Gillespie C., Rao C.V., Aldridge P.D. (2018). Driving the expression of the *Salmonella enterica* sv Typhimurium flagellum using *flhDC* from *Escherichia coli* results in key regulatory and cellular differences. Sci. Rep..

[23] Mekalanos J.J. (1992). Environmental signals controlling expression of virulence determinants in bacteria. J. Bacteriol..

[24] Soutourina O.A., Bertin P.N. (2003). Regulation cascade of flagellar expression in Gram-negative bacteria. FEMS Microbiol. Rev..

[25] McCarter L.L. (2006). Regulation of flagella. Curr. Opin. Microbiol..

[26] Lele P.P., Hosu B.G., Berg H.C. (2013). Dynamics of mechanosensing in the bacterial flagellar motor. Proc. Natl. Acad. Sci. USA.

[27] Spöring I., Felgner S., Preuße M., Eckweiler D., Rohde M., Häussler S., Weiss S., Erhardt M. (2018). Regulation of flagellum biosynthesis in response to cell envelope stress in *Salmonella enterica* serovar Typhimurium. mBio.

[28] Adler J., Templeton B. (1967). The effect of environmental conditions on the motility of *Escherichia coli*. J. Gen. Microbiol..

[29] Alm R.A., Guerry P., Trust T.J. (1993). The *Campylobacter sigma 54 flaB* flagellin promoter is subject to environmental regulation. J. Bacteriol..

[30] Harshey R.M. (2003). Bacterial motility on a surface: Many ways to a common goal. Annu. Rev. Microbiol..

[31] Maurer L.M., Yohannes E., Bondurant S.S., Radmacher M., Slonczewski J.L. (2005). pH regulates genes for flagellar motility, catabolism, and oxidative stress in *Escherichia coli* K-12. J. Bacteriol..

[32] Hockett K.L., Burch A.Y., Lindow S.E. (2013). Thermo-regulation of genes mediating motility and plant interactions in *Pseudomonas syringae*. PLoS ONE.

[33] Rudenko I., Ni B., Glatter T., Sourjik V. (2019). Inefficient secretion of anti-sigma factor FlgM inhibits bacterial motility at high temperature. iScience.

[34] Erdem A.L., Avelino F., Xicohtencatl-Cortes J., Girón J.A. (2007). Host protein binding and adhesive properties of H6 and H7 flagella of attaching and effacing *Escherichia coli*. J. Bacteriol..

[35] Wang T., Si M., Song Y., Zhu W., Gao F., Wang Y., Zhang L., Zhang W., Wei G., Luo Z.Q. (2015). Type VI secretion system transports Zn^2+^ to combat multiple stresses and host immunity. PLoS Pathog..

[36] Li X., Ren F., Cai G., Huang P., Chai Q., Gundogdu O., Jiao X., Huang J. (2020). Investigating the role of FlhF identifies novel interactions with genes involved in flagellar synthesis in *Campylobacter jejuni*. Front. Microbiol..

[37] Speare L., Woo M., Bultman K.M., Mandel M.J., Wollenberg M.S., Septer A.N. (2021). Host-Like conditions are required for T6SS-mediated competition among *Vibrio fischeri* light organ symbionts. mSphere.

[38] Allsopp L.P., Collins A.C.Z., Hawkins E., Wood T.E., Filloux A. (2022). RpoN/Sfa2-dependent activation of the *Pseudomonas aeruginosa* H2-T6SS and its cognate arsenal of antibacterial toxins. Nucleic Acids Res..

[39] Alvarez-Fraga L., Phan M.D., Goh K.G.K., Nhu N.T.K., Hancock S.J., Allsopp L.P., Peters K.M., Forde B.M., Roberts L.W., Sullivan M.J. (2022). Differential Afa/Dr fimbriae expression in the multidrug-resistant *Escherichia coli* ST131 clone. mBio.

[40] Miller J.H. (1972). Experiments in Molecular Genetics.

[41] Sperandio V., Torres A.G., Girón J.A., Kaper J.B. (2001). Quorum sensing is a global regulatory mechanism in enterohemorrhagic *Escherichia coli* O157:H7. J. Bacteriol..

[42] Schwan W.R., Lee J.L., Lenard F.A., Matthews B.T., Beck M.T. (2002). Osmolarity and pH growth conditions regulate *fim* gene transcription and type 1 pilus expression in uropathogenic *Escherichia coli*. Infect. Immun..

[43] Subramanian N., Qadri A. (2006). Lysophospholipid sensing triggers secretion of flagellin from pathogenic *salmonella*. Nat. Immunol..

[44] Castell D.O., Moore E.W. (1971). Ammonia absorption from the human colon. The role of nonionic diffusion. Gastroenterology.

[45] Ferraris R.P., Yasharpour S., Lloyd K.C., Mirzayan R., Diamond J.M. (1990). Luminal glucose concentrations in the gut under normal conditions. Am. J. Physiol. Liver Physiol..

[46] Mossberg S.M., Ross G. (1967). Ammonia movement in the small intestine: Preferential transport by the ileum. J. Clin. Investig..

[47] Abe H., Tatsuno I., Tobe T., Okutani A., Sasakawa C. (2002). Bicarbonate ion stimulates the expression of locus of enterocyte effacement-encoded genes in enterohemorrhagic *Escherichia coli* O157:H7. Infect. Immun..

[48] Elliott S.J., Sperandio V., Girón J.A., Shin S., Mellies J.L., Wainwright L., Hutcheson S.W., McDaniel T.K., Kaper J.B. (2000). The locus of enterocyte effacement (LEE)-encoded regulator controls expression of both LEE- and non-LEE-encoded virulence factors in enteropathogenic and enterohemorrhagic *Escherichia coli*. Infect. Immun..

[49] Puente J.L., Bieber D., Ramer S.W., Murray W., Schoolnik G.K. (1996). The bundle-forming pili of enteropathogenic *Escherichia coli*: Transcriptional regulation by environmental signals. Mol. Microbiol..

[50] Gismero-Ordoñez J., Dall’Agnol M., Trabulsi L.R., Girón J.A. (2002). Expression of the bundle-forming pilus by enteropathogenic *Escherichia coli* strains of heterologous serotypes. J. Clin. Microbiol..

[51] Kenny B., Abe A., Stein M., Finlay B.B. (1997). Enteropathogenic *Escherichia coli* protein secretion is induced in response to conditions similar to those in the gastrointestinal tract. Infect. Immun..

[52] Girón J.A., Ho A.S., Schoolnik G.K. (1991). An inducible bundle-forming pilus of enteropathogenic *Escherichia coli*. Science.

[53] Akerley B.J., Cotter P.A., Miller J.F. (1995). Ectopic expression of the flagellar regulon alters development of the *Bordetella*-host interaction. Cell.

[54] Schmitt C.K., Ikeda J.S., Darnell S.C., Watson P.R., Bispham J., Wallis T.S., Weinstein D.L., Metcalf E.S., O’Brien A.D. (2001). Absence of all components of the flagellar export and synthesis machinery differentially alters virulence of *Salmonella enterica* serovar Typhimurium in models of typhoid fever, survival in macrophages, tissue culture invasiveness, and calf enterocolitis. Infect. Immun..

[55] Lyte M., Arulanandam B., Nguyen K., Frank C., Erickson A., Francis D. (1997). Norepinephrine induced growth and expression of virulence associated factors in enterotoxigenic and enterohemorrhagic strains of *Escherichia coli*. Adv. Exp. Med. Biol..

[56] Sperandio V., Torres A.G., Jarvis B., Nataro J.P., Kaper J.B. (2003). Bacteria-host communication: The language of hormones. Proc. Natl. Acad. Sci. USA.

[57] Bansal T., Englert D., Lee J., Hegde M., Wood T.K., Jayaraman A. (2007). Differential effects of epinephrine, norepinephrine, and indole on *Escherichia coli* O157:H7 chemotaxis, colonization, and gene expression. Infect. Immun..

[58] Cotter P.A., Miller J.F. (1996). Triggering bacterial virulence. Science.

[59] De la Cruz M.A., Ares M.A., von Bargen K., Panunzi L.G., Martínez-Cruz J., Valdez-Salazar H.A., Jiménez-Galicia C., Torres J. (2017). Gene expression profiling of transcription factors of *Helicobacter pylori* under different environmental conditions. Front. Microbiol..

[60] Ares M.A., Abundes-Gallegos J., Rodríguez-Valverde D., Panunzi L.G., Jiménez-Galicia C., Jarillo-Quijada M.D., Cedillo M.L., Alcántar-Curiel M.D., Torres J., Girón J.A. (2019). The coli surface antigen CS3 of Enterotoxigenic *Escherichia coli* is differentially regulated by H-NS, CRP, and CpxRA global regulators. Front. Microbiol..

[61] Do H., Makthal N., VanderWal A.R., Saavedra M.O., Olsen R.J., Musser J.M., Kumaraswami M. (2019). Environmental pH and peptide signaling control virulence of *Streptococcus pyogenes* via a quorum-sensing pathway. Nat. Commun..

[62] Rodríguez-Valverde D., León-Montes N., Soria-Bustos J., Martínez-Cruz J., González-Ugalde R., Rivera-Gutiérrez S., González-Y.-Merchand J.A., Rosales-Reyes R., García-Morales L., Hirakawa H. (2021). cAMP receptor protein positively regulates the expression of genes involved in the biosynthesis of *Klebsiella oxytoca* tilivalline cytotoxin. Front. Microbiol..

[63] Valdez-Salazar H.A., Ares M.A., Fernández F.J., Ibarra J.A., Torres J., Bustamante V.H., De la Cruz M.A. (2021). Long-chain fatty acids alter transcription of *Helicobacter pylori* virulence and regulatory genes. PeerJ.

[64] Leverton L.Q., Kaper J.B. (2005). Temporal expression of enteropathogenic *Escherichia coli* virulence genes in an in vitro model of infection. Infect. Immun..

[65] Ronin I., Katsowich N., Rosenshine I., Balaban N.Q. (2017). A long-term epigenetic memory switch controls bacterial virulence bimodality. eLife.

[66] Platenkamp A., Mellies J.L. (2018). Environment controls LEE regulation in enteropathogenic *Escherichia coli*. Front. Microbiol..

[67] Walker S.L., Sojka M., Dibb-Fuller M., Woodward M.J. (1999). Effect of pH, temperature and surface contact on the elaboration of fimbriae and flagella by *Salmonella* serotype Enteritidis. J. Med. Microbiol..

[68] Zhao K., Liu M., Burgess R.R. (2007). Adaptation in bacterial flagellar and motility systems: From regulon members to ‘foraging’-like behavior in *E. coli*. Nucleic Acids Res..

[69] Wang F., Deng L., Huang F., Wang Z., Lu Q., Xu C. (2020). Flagellar motility is critical for *Salmonella enterica* serovar Typhimurium biofilm development. Front. Microbiol..

[70] Yang J., Hart E., Tauschek M., Price G.D., Hartland E.L., Strugnell R.A., Robins-Browne R.M. (2008). Bicarbonate-mediated transcriptional activation of divergent operons by the virulence regulatory protein, RegA, from *Citrobacter rodentium*. Mol. Microbiol..

[71] Abuaita B.H., Withey J.H. (2009). Bicarbonate Induces *Vibrio cholerae* virulence gene expression by enhancing ToxT activity. Infect. Immun..

[72] Migliore F., Macchi R., Landini P., Paroni M. (2018). Phagocytosis and epithelial cell invasion by Crohn’s disease-associated adherent-invasive *Escherichia coli* are inhibited by the anti-inflammatory drug 6-Mercaptopurine. Front. Microbiol..

[73] Bijlsma J.J., Groisman E.A. (2005). The PhoP/PhoQ system controls the intramacrophage type three secretion system of *Salmonella enterica*. Mol. Microbiol..

[74] De la Cruz M.A., Ruiz-Tagle A., Ares M.A., Pacheco S., Yáñez J.A., Cedillo L., Torres J., Girón J.A. (2017). The expression of Longus type 4 pilus of enterotoxigenic *Escherichia coli* is regulated by LngR and LngS and by H-NS, CpxR and CRP global regulators. Environ. Microbiol..

[75] Liu Y., Han R., Wang J., Yang P., Wang F., Yang B. (2020). Magnesium sensing regulates intestinal colonization of Enterohemorrhagic *Escherichia coli* O157:H7. mBio.

[76] Deng W., Puente J.L., Gruenheid S., Li Y., Vallance B.A., Vázquez A., Barba J., Ibarra J.A., O’Donnell P., Metalnikov P. (2004). Dissecting virulence: Systematic and functional analyses of a pathogenicity island. Proc. Natl. Acad. Sci. USA.

[77] Gaytán M.O., Monjarás Feria J., Soto E., Espinosa N., Benítez J.M., Georgellis D., González-Pedrajo B. (2017). Novel insights into the mechanism of SepL-mediated control of effector secretion in enteropathogenic *Escherichia coli*. Microbiologyopen.

[78] Shaulov L., Gershberg J., Deng W., Finlay B.B., Sal-Man N. (2017). The ruler protein EscP of the enteropathogenic *Escherichia coli* type III secretion system is involved in calcium sensing and secretion hierarchy regulation by interacting with the gatekeeper protein SepL. mBio.

[79] Yarwood J.M., Schlievert P.M. (2000). Oxygen and carbon dioxide regulation of toxic shock syndrome toxin 1 production by *Staphylococcus aureus* MN8. J. Clin. Microbiol..

[80] Ramos-Morales F. (2012). Acidic pH: Enemy or ally for enteric bacteria?. Virulence.

[81] Danielsson H. (1963). Influence of bile acids on digestion and absorption of lipids. Am. J. Clin. Nutr..

[82] Chiang J.Y.L., Ferrell J.M. (2019). Bile acids as metabolic regulators and nutrient sensors. Annu. Rev. Nutr..

[83] Hung D.T., Mekalanos J.J. (2005). Bile acids induce cholera toxin expression in *Vibrio cholerae* in a ToxT-independent manner. Proc. Natl. Acad. Sci. USA.

[84] Hung D.T., Zhu J., Sturtevant D., Mekalanos J.J. (2006). Bile acids stimulate biofilm formation in *Vibrio cholerae*. Mol. Microbiol..

[85] Yang M., Liu Z., Hughes C., Stern A.M., Wang H., Zhong Z., Kan B., Fenical W., Zhu J. (2013). Bile salt-induced intermolecular disulfide bond formation activates *Vibrio cholerae* virulence. Proc. Natl. Acad. Sci. USA.

[86] D’Mello A., Yotis W.W. (1987). The action of sodium deoxycholate on *Escherichia coli*. Appl. Environ. Microbiol..

[87] Lyte M. (2004). Microbial endocrinology and infectious disease in the 21st century. Trends Microbiol..

[88] Cambronel M., Nilly F., Mesguida O., Boukerb A.M., Racine P.J., Baccouri O., Borrel V., Martel J., Fécamp F., Knowlton R. (2020). Influence of catecholamines (Epinephrine/Norepinephrine) on biofilm formation and adhesion in pathogenic and probiotic strains of *Enterococcus faecalis*. Front. Microbiol..

[89] Alverdy J., Holbrook C., Rocha F., Seiden L., Wu R.L., Musch M., Chang E., Ohman D., Suh S. (2000). Gut-derived sepsis occurs when the right pathogen with the right virulence genes meets the right host: Evidence for in vivo virulence expression in *Pseudomonas aeruginosa*. Ann. Surg..

[90] Cogan T.A., Thomas A.O., Rees L.E., Taylor A.H., Jepson M.A., Williams P.H., Ketley J., Humphrey T.J. (2007). Norepinephrine increases the pathogenic potential of *Campylobacter jejuni*. Gut.

[91] Walters M., Sperandio V. (2006). Autoinducer 3 and epinephrine signaling in the kinetics of locus of enterocyte effacement gene expression in enterohemorrhagic *Escherichia coli*. Infect. Immun..

[92] Beata S., Michał T., Mateusz O., Urszula W., Choroszy M., Andrzej T., Piotr D. (2021). Norepinephrine affects the interaction of adherent-invasive *Escherichia coli* with intestinal epithelial cells. Virulence.

